# Age and Sex of Mice Markedly Affect Survival Times Associated with Hyperoxic Acute Lung Injury

**DOI:** 10.1371/journal.pone.0130936

**Published:** 2015-06-23

**Authors:** Daniel R. Prows, William J. Gibbons, Jessica J. Smith, Valentina Pilipenko, Lisa J. Martin

**Affiliations:** 1 Division of Human Genetics, Cincinnati Children’s Hospital Medical Center, Cincinnati, Ohio, United States of America; 2 Department of Pediatrics, University of Cincinnati College of Medicine, Cincinnati, Ohio, United States of America; 3 Division of Biostatistics & Epidemiology, Cincinnati Children’s Hospital Medical Center, Cincinnati, Ohio, United States of America; Children's Hospital Los Angeles, UNITED STATES

## Abstract

Mortality associated with acute lung injury (ALI) remains substantial, with recent estimates of 35–45% similar to those obtained decades ago. Although evidence for sex-related differences in ALI mortality remains equivocal, death rates differ markedly for age, with more than 3-fold increased mortality in older versus younger patients. Strains of mice also show large differences in ALI mortality. To tease out genetic factors affecting mortality, we established a mouse model of differential hyperoxic ALI (HALI) survival. Separate genetic analyses of backcross and F_2_ populations generated from sensitive C57BL/6J (B) and resistant 129X1/SvJ (X1) progenitor strains identified two quantitative trait loci (QTLs; *Shali1* and *Shali2*) with strong, equal but opposite, within-strain effects on survival. Congenic lines confirmed these opposing QTL effects, but also retained the low penetrance seen in the 6–12 week X1 control strain. Sorting mice into distinct age groups revealed that ‘age at exposure’ inversely correlated with survival time and explained reduced penetrance of the resistance trait. While B mice were already sensitive by 6 weeks old, X1 mice maintained significant resistance up to 3–4 weeks longer. Reanalysis of F_2_ data gave analogous age-related findings, and also supported sex-specific linkage for *Shali1* and *Shali2*. Importantly, we have demonstrated in congenic mice that these age effects on survival correspond with B alleles for *Shali1* (6-week old mice more sensitive) and *Shali2* (10-week old mice more resistant) placed on the X1 background. Further studies revealed significant sex-specific survival differences in subcongenics for both QTLs. Accounting for age and sex markedly improved penetrance of both QTLs, thereby reducing trait variability, refining *Shali1* to <8.5Mb, and supporting several sub-QTLs within the *Shali2* interval. Together, these congenics will allow age- and sex-specific studies to interrogate myriad subphenotypes affected during ALI development and progression and identify intermediary injury biomarkers that can predict outcome.

## Introduction

Acute lung injury (ALI) and acute respiratory distress syndrome (ARDS) represent a continuum of a rapidly progressing, life-threatening disease, defined by diffuse alveolar damage, pulmonary edema, severe hypoxemia, and high mortality. Despite decades of focused research, death rates associated with ALI still exceed 35% in most clinical settings [1]. With over 100,000 deaths per year [2], ALI mortality is higher than that for AIDS, asthma, breast cancer or myocardial infarction [3]. Mortality rates range from > 60% in the elderly to < 20% in children [2], indicating the geriatric population is a highly susceptible group [4–6]; in fact, advanced age is an independent predictor of ALI death. With an aging population, ALI incidence was recently estimated to reach 335,000 cases per year in the US by 2030, leading to ~147,000 deaths annually [2]. Worldwide figures show that ALI is widespread, with massive socio-economic impact. These projections, along with stalled progress to further reduce ALI mortality rates, highlight a need for alternative research strategies to curb a potential healthcare crisis as the world population continues to age.

Our long-term goal is to reduce morbidity and mortality associated with ALI. Accordingly, we have established a mouse model of differential ALI survival using hyperoxia [7–10], a classical oxidant injury often evaluated for its hallmark features of ALI in animals [11]. An initial screen of 6–12 week old females for 18 inbred mouse strains revealed that C57BL/6J inbred mice (B) had short survival times (*i*.*e*., sensitive), but 129X1/SvJ inbred mice (X1) survived considerably longer (*i*.*e*., resistant) [9]. Although penetrance of the prolonged resistance in X1 mice was only about 30–35%, two separate QTL analyses of large segregating populations (*i*.*e*., 840 F_2_ and 935 N_2_ backcross mice) generated from the B and X1 progenitor strains both identified the same two highly significant QTLs (*Shali1* on Chr1 and *Shali2* on Chr4; designated *S*urvival time with *h*yperoxic *a*cute *l*ung *i*njury) linked to differential survival time [8,10]. *In silico* analyses predicted that the major allelic effects of *Shali1* and *Shali2* on survival time opposed each other: X1 alleles of *Shali1* and B alleles of *Shali2* prolonged survival, whereas B alleles of *Shali1* and X1 alleles of *Shali2* reduced survival times. These opposing QTL effects were both confirmed in separate congenic lines on the X1 background [7]. Penetrance of the sensitivity trait on Chr1 is high in *Shali1* congenics and therefore conducive to further refinement and gene discovery. The low penetrance of the resistance trait on Chr4, however, has persisted in *Shali2* congenics, heretofore making further resolution of *Shali2* considerably more problematic.

In this report, we show that *Shali1* and *Shali2* effects on survival directly correlate with age of mice at the start of hyperoxia exposures. In particular, highly significant differences in mean survival times (MSTs) were associated with a ~3 to 4-week period in young adulthood. Although X1 is designated a resistant strain, we discovered that this resistance is highly age-dependent. In particular, six-week old X1 mice survived hyperoxia-induced acute lung injury (HALI) about twice as long as 10-week old X1 mice. This age-related change accounted for much of the reduced penetrance of the resistance trait in X1 mice (originally exposed at 6–12 weeks old), and variable penetrance of resistance seen in many X1-derived congenic lines. Thus, the *Shali1* and *Shali2* intervals house important genes associated with the sensitivity and resistance traits, respectively. Congenic lines for the two QTLs have separately confirmed these opposing age- and sex-specific effects: B-alleles for *Shali1* leads to increased sensitivity (earlier death) with highest penetrance in 6-week females, and B-alleles for *Shali2* impart increased resistance (prolonged survival) with highest penetrance in 10-week mice of either or both sexes, depending on the specific *Shali2* subcongenic line. The age of this transition period correlates with the completion of lung alveolarization and sexual maturation, suggesting either or both processes are significant confounders worthy of additional studies. Using age and sex for testing and screening congenics has narrowed *Shali1* to ~8.5 Mb and provided evidence for several sub-QTLs for *Shali2*. Together, the corresponding congenics should allow us to resolve these *Shali* QTLs and to further examine important genetic or epigenetic factors at play. These mice also provide the tools to critically investigate intermediary traits of HALI and other models of ALI during the development and progression of lung injury, with potential to identify common and age- or sex-specific biomarkers that can help prognose ALI severity or outcome.

## Materials and Methods

### Mice

The protocol for the use of mice in these studies was approved by the Institutional Animal Care and Use Committee at Cincinnati Children’s Hospital Research Foundation. Because sensitivity versus resistance alleles resulted in significant differences in survival times and the reduced penetrance was an additional confounder, a continuous and uninterrupted exposure was required for valid results. Protocol #IACUC2013-0021 was approved for survival studies with death as an endpoint for up to 14 days, at which point any remaining mice were euthanized by CO_2_ asphyxiation. The sensitive C57BL/6J (B) and resistant 129X1/SvJ (X1) inbred mouse strains were purchased from the Jackson Laboratory (Bar Harbor, ME) and used directly in exposures at 6–12 weeks of age as controls. Inbred mice were also used for in-house breeding of control lines and in the construction of congenics and subcongenics for *Shali1* and *Shali2* on chromosomes (Chrs) 1 and 4, respectively. When bred in-house, B and X1 mice were replaced after five brother-sister generations to minimize genetic drift in the colony.

### Trait of interest

The long-term goal of this project is to identify specific genes or pathways beneficial for increased survival to oxidant-induced lung injury. Accordingly, survival time was selected as the trait of interest, because the high mortality rate from ALI is the ultimate outcome that must be improved. We are the first investigators to identify (through genetic linkage analysis) chromosomal regions harboring genes that affect ALI survival time when induced by oxidative agents [8,12,13]. Importantly, the genetic linkage from our survival studies differs from those for genetic studies of other intermediate traits. In fact, genetic studies that assess earlier indicators of ALI in mice (*e*.*g*., subphenotypes such as lung histology, inflammation, and protein or leukocytes in bronchoalveolar lavage fluid) have identified different chromosomal regions linked to those traits [14–17]. Thus, these intermediate phenotypes do not correlate with outcome and strongly suggest that the genes important for survival/mortality differ from those causing earlier morbidities. Current data from these mouse studies also agrees with findings in the clinical literature; *i*.*e*., no early or intermediate trait (*e*.*g*., gene/protein/biomarker) reliably predicts ALI outcome. Our strategy is to directly identify genes linked to survival differences, which can provide new candidate genes and pathways towards improving mortality rates.

### Construction of new congenic and subcongenic lines

Congenic and subcongenic lines described in these studies were generated from our initial consomics and congenic lines [7], and are all maintained on the X1 background. Herein, lines for *Shali1* and *Shali2* are designated as X1.B-1*x* and X1.B-4*x*, respectively; X1 = background strain (129X1/SvJ), followed by the substituted Chr strain B (C57BL/6J) and the substituted Chr number (1 or 4); *x* denotes the specific congenic or subcongenic line. Congenics and subcongenics were produced using a backcross-intercross breeding scheme. Panels of nested congenic lines were made using microsatellites to screen the QTL intervals. Each crossover was reproduced by backcrossing to the X1 strain to generate a breeder mate, and the congenic regions were fixed to homozygosity for testing and maintenance of lines. A genomewide analysis using the MUGA Mouse SNP panel was performed (GeneSeek; Lincoln, NE), after which microsatellite markers or individual SNPs were used to directly target the removal of any remaining heterozygous regions in the genome or in the congenic interval. Thus, these lines are true congenics to the resolution of the MUGA panel for X1 versus B strains.

### DNA preparation

Genomic DNA was isolated from 2–3 mm tail tips using Wizard Genomic DNA kit (Promega; Madison, WI). Purity (A_260_/A_280_) and DNA content (A_260_) were assessed using a Nanodrop 8000 (Thermo Fisher Scientific; Waltham MA). For PCR requiring agarose gel separation, DNA was diluted to ~20 ng/μl. Genotyping of fluorescent-labeled markers used DNA diluted to ~5 ng/μl for PCR.

### Genotype analysis

Primer pairs for microsatellite markers polymorphic between the B and X1 strains were purchased from IDT (Coralville, IA). PCR (15-μL reactions) was performed in 96-well plates (USA Scientific; Ocala, FL) using a 4-block thermocycler (BioRad, Model PTC-225 or PTC-240), as described previously [7,8]. Markers with allele sizes ≥ 5% were separated with gels made of 2.5–3.5% agarose (BioExpress; Kaysville, UT). Final band sizes were discriminated by ethidium bromide staining. Microsatellite markers with allele sizes differing less than 5% were amplified using fluorescent primers synthesized at Applied Biosystems by Life Technologies (now part of ThermoFisher Scientific, Waltham, MA). Fluorescent PCR products of microsatellites markers were separated using an ABI-3730*xL* sequencer located at the Cincinnati Children’s Genetic Variation and Gene Discovery Core (http://dna.cchmc.org/), and genotypes ascertained using GeneMapper software (V4.0, ABI). For any remaining regions identified as heterozygous by the MUGA or MegaMUGA SNP panels (GeneSeek, Inc.), targeted microsatellite markers were used whenever possible to remove undesired B-alleles or to fix them in a homozygous state. For small regions with no known polymorphic microsatellite markers, individual SNPs (ABI) were used with real-time qPCR (ABI 7300).

### Hyperoxia exposures

Mice were kept in their standard shoebox cages (food and water *ad libitum*) and placed within a 0.13-m^3^ Plexiglas inhalation chamber (manufactured to specifications by Stellar Plastics, Detroit, MI) and exposed continuously to > 95% O_2_ until death. Individual mice were clearly visible from above and/or through sides of the chamber and cages to assess status. Each chamber could house up to nine shoebox cages and each cage up to four mice. During exposures, males from different litters were not housed together and females were not placed with males at less than a 1:1 ratio. The chamber O_2_ level was continuously monitored using ProOx 110 (Biospherix, Redfield, NY) or battery-operated MiniOx 3000 (MSA, Pittsburgh, PA) O_2_ monitors. Status of mice and the exposure conditions were closely observed, such that the time between measures was within 5% error of the overall exposure to that point. The survival time used for each mouse was the average between the previous time check and the time the mouse was identified as dead. Up until the age-related effect was identified, mice were exposed between 6 and 12 weeks of age. After the age effect was discovered, lines were primarily exposed at ~6 weeks (*i*.*e*., 42 ± 3 days old; 39–45 d.o.) or ~10 weeks (70 ± 3 d.o) of age. To prevent hypoxic seizures and/or premature deaths upon opening the chamber (which would invalidate the times of any deaths thereafter), each exposure was continued uninterrupted until all mice within the chamber succumbed.

### Age- and sex-specific analyses

The high variability seen in the survival times among genetically identical mice suggested other factors were involved in the differential response to HALI. We therefore examined the data for possible interactions for strain, sex, and/or age. For the two control strains, a 3-way interaction for strain by sex by age did not show significance. Similarly, 2-way interactions for sex by age and sex by strain also did not reach significance. However, a 2-way interaction of age by strain (sex as a covariate) was significant. These same interaction models were run for the F_2_ dataset, which identified a significant interaction for sex by age. These findings supported the need to include strain, age and sex in all analyses.

#### Age

Initially, all exposed mice for survival studies ranged between 6–12 weeks of age. To assess the possibility that age might play a role in differential survival times the X1 and B inbred strains, congenic and subcongenic lines, and all F_2_ mice of the original QTL analysis [8] were re-evaluated. Because of their use as controls over many years of exposures, cohort sizes of inbred strains were large enough to sort the data into 1-week age bins. Based on results from these control strains, initial congenic lines were tested in hyperoxia at larger numbers and sorted into groups of mice at ≤ 6 weeks, 7–9 weeks, and ≥ 10 weeks old for analysis of an age effect. To determine whether the age effect associated with survival time in the original F_2_ population (n = 840), this dataset was sorted into the same 3 age groups for comparisons.

#### Sex

Our initial report for the HALI mouse model [8] noted a small, yet significant, survival difference between males and females of the B strain; this sex-effect was not evident in the X1 strain at that time. However, sex was not accounted for in these earlier analyses. Because analysis of the full F_2_ dataset supported a significant age by sex interaction, we further assessed whether a sex-effect was present in these newly defined age-specific groups for controls, congenics and F_2_ cohorts. To address the expected small group sizes when parsing of the F_2_ data by genotype, age, and sex, the total F_2_ population was re-sorted into “younger” and “older” cohorts using a threshold age of ≤ 56 d.o.; this age represented the exact mid-point of the critical 4-week trait transition period of 6–10 weeks (*i*.*e*., 42–70 d.o.). QTL analysis was then performed separately on these two age-defined F_2_ groups (*i*.*e*., younger *vs*. older), with further separate analyses for total mice and both sexes.

### Statistical analyses

Prior to statistical inference testing, we evaluated the distribution of MST. As MST was sufficiently normally distributed, MSTs and SDs are presented. To determine whether there were age, sex, and strain effects on MST, groups were compared using the Student’s *t*-test. To account for the possibility of sex, age and strain effects we first separated the controls into 12 subgroups, creating unique combinations of age (3 groups), sex (2 groups) and strain (2 groups). For age, each of the three age categories was compared to each other within the four sex and strain cohorts. Because of concerns of increased false positives, we used the Bonferroni adjusted significance threshold for age comparisons (0.05/12; *p* ≤ 0.00417). *T*-tests were also performed for the sex and strain groups using the same strategy. To account for multiple testing for sex and strain the significant threshold was *p* ≤ 0.0083 (0.05/6) after Bonferroni correction. To determine whether age interacted with sex or strain linear regression models were used. For all models, age, sex, and strain were included as main effects. A single interaction term (either age by sex or age by strain) was also included. Significance for interaction terms accounted for the two tests, *p* ≤ 0.025 (0.05/2).

The F_2_ dataset [8] was reanalyzed in several ways. First, the total population (n = 840) was split by sex and age group (≤ 6 weeks, 7–9 weeks, and ≥ 10 weeks old), and MSTs calculated and evaluated for differences using *t*-tests with multi-test corrections. Linear regression was used to evaluate age by sex interactions. Next, the total dataset of exposed F_2_ mice was stratified by genotypes at the peak markers for *Shali1* and *Shali2*, with interest only in the two cohorts of mice carrying homozygosity for both resistance alleles (X1-B, n = 50) or both sensitivity alleles (B-X1, n = 58) at *D1Mit303* and *D4Mit308*, respectively. Each of these genotype groups was then parsed by age (younger or older) using 56 d.o. as the discriminator. To assess a possible sex effect, each of these four age groups was then divided into males and females. Groups were assessed for significant differences by *t*-tests with the appropriate Bonferroni corrections for multiple comparisons. In a third analysis, the F_2_ dataset was divided into younger *versus* older aged cohorts using a single-age threshold of 56 d.o. and separate QTL analyses were performed for both sexes in each age group using R/QTL [18].

## Results

### MSTs Vary by Age and Sex in the X1 and B Inbred Control Strains

**Fig 1:**
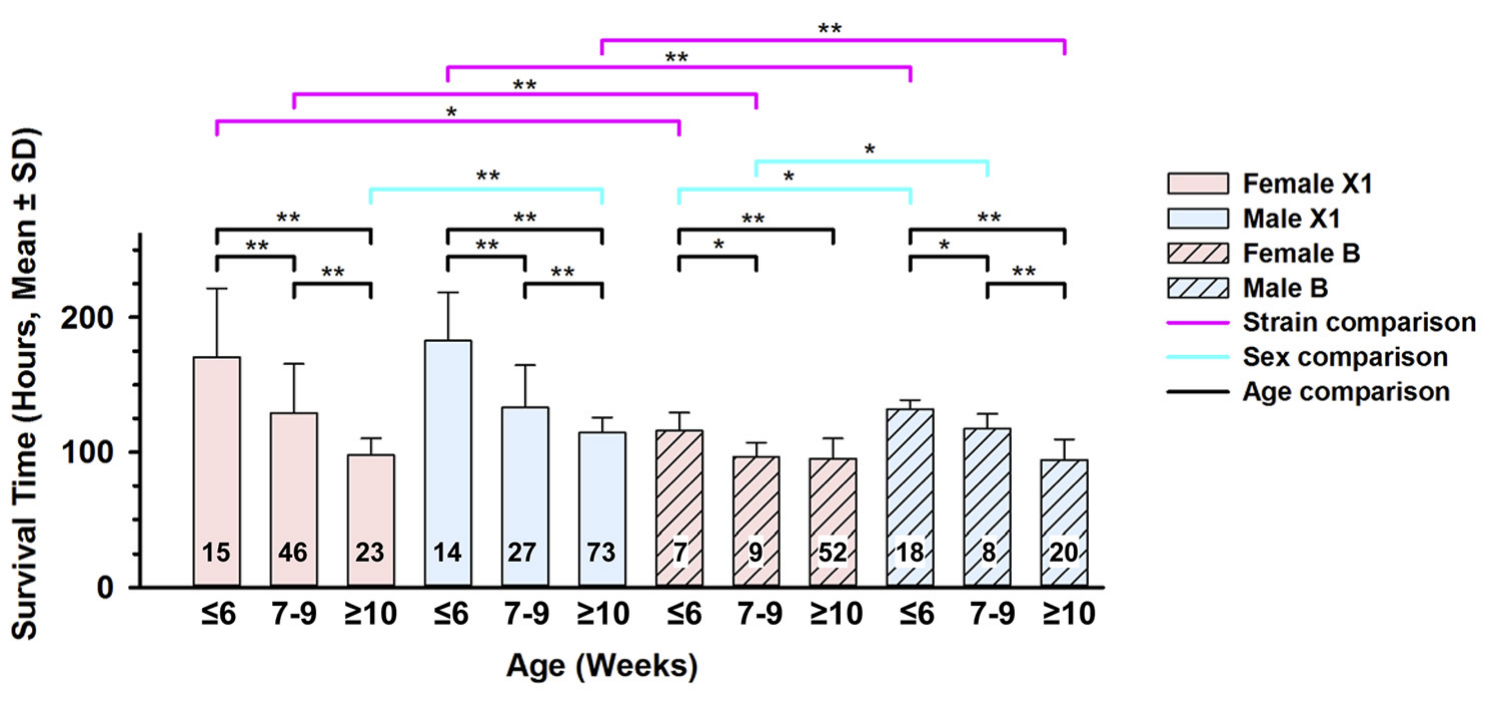
Survival time comparisons of X1 and B controls stratified by age and sex. Survival times for all exposed mice (6–12 weeks old) of each progenitor strain were combined and sorted by sex into three distinct age groups: ≤ 6 weeks old, 7–9 weeks old, and ≥ 10 weeks old at the initiation of exposure. Resistant X1 control mice (solid bars) are displayed at the left half of the figure, with sensitive B control mice (striped bars) shown at the right half. The number of mice tested in each group is given at the base of each bar plot. For direct within-strain sex comparisons, females (pink bars) and males (blue bars) are positioned side-by-side. Comparisons were also made between age groups (females or males) and between strains for the same age group, with Bonferroni corrections for multiple testing. *, p < 0.05; **, p < 0.001.

Previous studies had exposed all mice to > 95% O_2_ within a narrow 6-week window during young adulthood, expecting that this narrow age window would reduce heterogeneity. However, we observed considerable variability in MSTs, leading us to explore possible reasons for this variability. After stratifying control mice into 12 subgroups of age (3 groups), sex (2 groups) and strain (2 groups), we found statistically significant differences between the oldest (≥ 10 weeks) and youngest (≤ 6 weeks) age groups across each of the four sex/strain categories (*i*.*e*., female X1, male X1, female B and male B), with older mice of both sexes and both strains showing the most sensitivity (Fig 1). The four intermediate age groups (7–9 weeks old) showed statistically significant differences from older and younger groups of the same sex and strain, except for intermediate age B females, which were more sensitive than younger B females (*p* = 0.0097), but did not differ from older B females. In addition, sex differences were present in older X1 mice (*p* < 0.0001), and younger (*p* < 0.0188) and mid-aged (*p* < 0.0011) B mice; in all these comparisons, males were more resistant than females. Because there also appeared to be marked differences in the effect of age between the sex/strain groups, we then performed linear regression analysis to test for the presence of age by sex and age by strain interactions. While the age by sex interaction was not statistically significant (*p* = 0.7924), the age by strain effect was highly significant (*p* < 0.0001).

### MSTs Vary by Age and Sex in the F_2_ population

**Fig 2:**
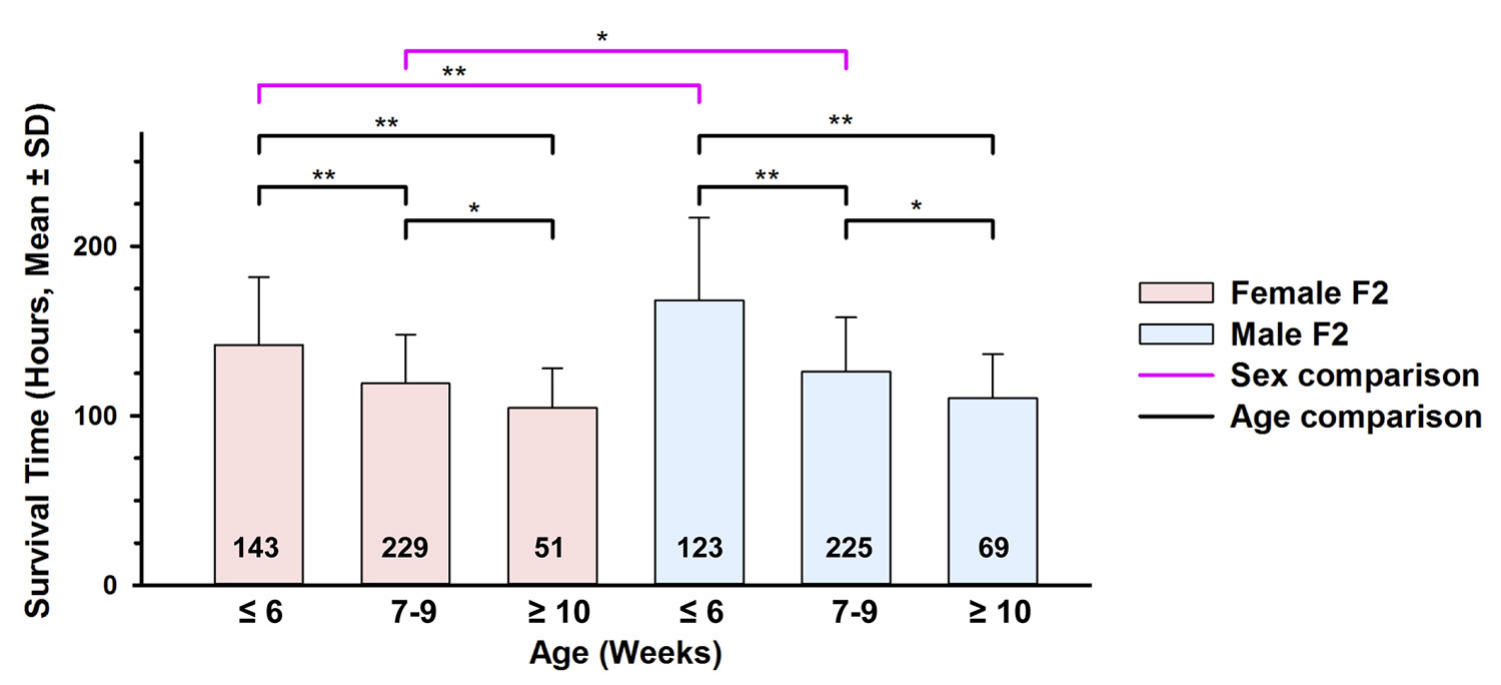
Survival time comparisons of F_2_ mice stratified by age and sex. Survival times for all exposed F_**2**_ mice (n = 840) generated from B and X1 progenitors, were sorted by sex into the three age groups: ≤ 6 weeks old, 7–9 weeks old, and ≥10 weeks old at the initiation of exposure. The number of F_**2**_ mice represented in each group is given at the base of each bar plot. Comparisons were made between age groups for females or males and between sexes for the same age group, and Bonferroni corrected for multiple testing. *, p < 0.05; **, p < 0.001.

Consistent with the parental strain effects, both the age and sex effects were also present in the (X1xB) F_2_ generation (Fig 2). Younger F_2_ males were significantly (*p* < 0.0001) more resistant (MST = 168 h; n = 123) than younger F_2_ females (MST = 142 h; n = 143) and intermediate age males were more resistant than intermediate age females (*p* = 0.014). Older F_2_ males and females were not significantly different, but there was still a trend towards increased male resistance. Interesting, the MST of F_2_ males or females in all age groups were similar to the average of the MSTs of X1 and B mice for the same sex and age group. For example, the MST of young F_2_ females was nearly identical to the average of MSTs for the X1 and B females (*i*.*e*., 142 h *vs*. 143 h). Similarly, the MST of young F_2_ males approximated the average of MSTs for X1 and B males (*i*.*e*., 168 h *vs*. 157 h). When performing formal interaction analyses, we found statistically significant evidence of age by sex interaction in the F_2_ generation (*p* = 0.002). These age and sex differences, along with age by sex interaction effects in the F_2_ generation are important, because this is the same population of mice used to localize the *Shali* QTLs. Presence of interaction suggested that sex- and age-stratified QTL analyses may be required to maximize power.

### QTL analysis of age and sex-specific F_2_ cohorts

**Fig 3:**
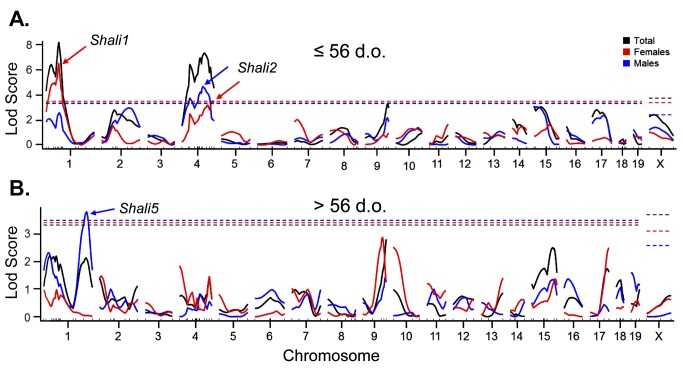
QTL analysis of age and sex cohorts of the total (X1xB) F_2_ dataset. The total F_**2**_ data set was sorted using a 56 d.o. age threshold for younger versus older cohorts and QTL analysis performed for total mice (black lines), and separately for males (blue lines) and females (red lines) in each age group. **A**. Younger mice (≤ 56 d.o.; n = 546) identified *Shali1* and *Shali2*, with significant differences between males (blue arrow) and females (red arrows) for each QTL. **B**. Older mice (> 56 d.o.; n = 294) did not identify *Shali1* or *Shali2*, but instead identified the male-specific *Shali5* on distal Chr1 (blue arrow).

The F_2_ dataset was next examined for putative age-related linkage to survival time using a single-age threshold of 56 d.o. (8 weeks) to sort mice into “younger” and “older” cohorts; this age divided the 6–10 week trait transition period exactly in half. Table 1 provides the descriptive statistics and Fig 3 the QTL analyses results for these two age groups, including total F_2_ mice (black lines), males (blue lines) and females (red lines). The QTL analysis plots of all F_2_ mice ≤ 56 d.o. at exposure (n = 546) is shown in Fig 3A. Results were similar to the full F_2_ dataset of 6–12 week old mice [8], and reaffirmed *Shali1* and *Shali2* as important putative QTLs for survival time differences. *Shali3* (Chr15) and *Shali4* (Chr9) from our initial findings on the full F_2_ population both fell just below the significance threshold for the ≤ 56 d.o. cohorts (Fig 3A). Interestingly, QTL analysis of all F_2_ mice > 56 d.o. (n = 294) did not identify any significant linkages (Fig 3B, black lines). Re-analysis after removing variance explained by ‘age at exposure’ found no additional significant loci.

**Table 1:** Survival times (hours) of F_2_ mice stratified by sex and 8-week age threshold.

Age Group	Total F_2_ population			Females			Males		
	n	Mean	SD	n	Mean	SD	n	Mean	SD
≤ 8 weeks (≤ 56 d.o.)	546	140.6	41.7	290	133.4	35.9	256	148.8	46.3
> 8 weeks (> 56 d.o.)	294	111.8	25.6	133	107.4	25.0	161	115.5	25.6

Next, the two age-specific F_2_ cohorts were stratified by sex for separate QTL analyses. Younger males (n = 256) yielded a significant linkage peak (Fig 3A, blue arrow) at *Shali2*, whereas younger females (n = 290) were highly significant for *Shali1* and *Shali2* (Fig 3A, red arrows). Of note, the male and female linkage peaks for *Shali2* (blue and red arrows, respectively) do not represent the same mapping locations on Chr4. No other significant QTLs were found in these younger cohorts. For older males (n = 161), only the previously identified male-specific QTL, *Shali5* on distal Chr1, was identified (Fig 3B, blue arrow). Analysis of older females (n = 133) found no significant linkage (Fig 3B). Thus, QTL analysis supported that differential survival time in hyperoxia between B and X1 mice can be defined by the age- and sex-related effects linked to both *Shali1* and *Shali2*, with support for additional follow up studies for the male-specific *Shali5* locus on distal Chr1.

### Genotype, age, and sex effects in F_2_ mice

**Fig 4:**
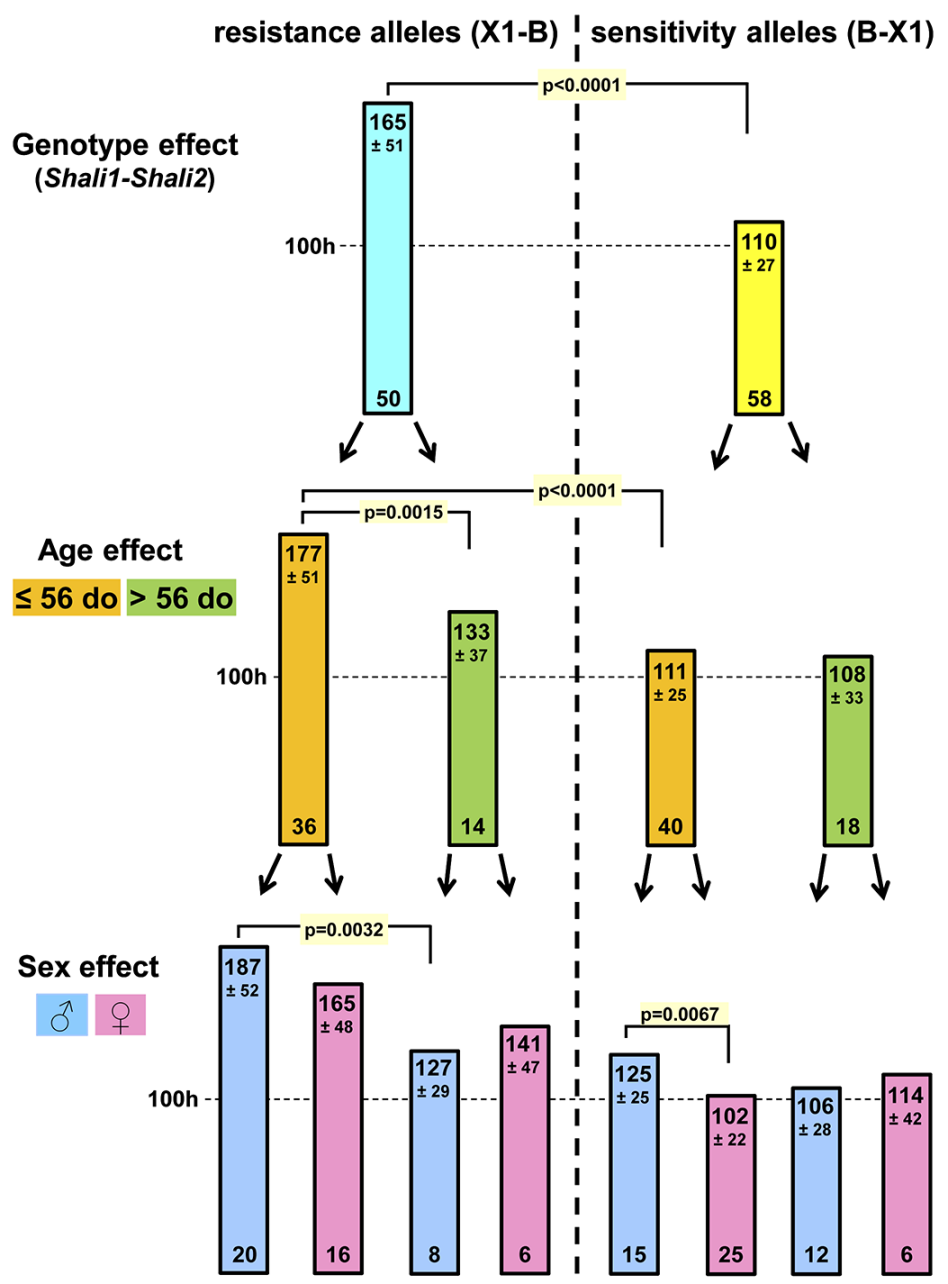
Age and sex-specific survival differences of F_2_ mice carrying reciprocal alleles for *Shali1* and *Shali2*. The (X1xB) F_**2**_ dataset (n = 840) was sorted based on resistance (X1-B) or sensitivity (B-X1) alleles (*i*.*e*., genotype effect) at both of the peak markers representing *Shali1* (*D1Mit303*) and *Shali2* (*D4Mit308*). Mice with the most resistant (X1-B; n = 50) and most sensitive (B-X1; n = 58) allelic combinations at these two loci were then segregated by age (using a 56 d.o. threshold for younger vs. older mice) and sex, and compared at each stage. Numbers near the top of each bar represent mean survival times (hours) and SDs rounded to the nearest hour, and those at the bottom of bars are numbers of mice with the corresponding variables (genotype/age/sex). Significant differences are as indicated, using the *t*-test for group comparisons and Bonferroni corrections for multiple testing.

Having previously shown that F_2_ mice carrying resistance alleles (n = 50) or sensitivity alleles (n = 58) for both *Shali1* and *Shali2* have the corresponding additive effects on HALI survival time [7], we next examined whether these cohorts of F_2_ mice also showed differential effects based on age and/or sex. Mice carrying these reciprocal allelic pairs (*i*.*e*., X1X1–BB *vs*. BB–X1X1 for *Shali1–Shali2*, respectively) demonstrated significant survival differences (*p* < 0.0001), with MSTs differing by 50% (Fig 4). Using 56 d.o. as the age threshold to stratify mice with the combined resistance and sensitivity alleles into younger and older cohorts, we again found a significant age-related effect, but only in the group of mice carrying the resistance alleles. Younger mice (MST = 177 h; n = 36) with resistance alleles at *Shali1* and *Shali2* were 33% more resistant (*p* = 0.0015) than older mice (MST = 133 h; n = 14) carrying the same alleles. In addition, younger mice carrying the *Shali1–Shali2* resistance alleles were dramatically (*p* < 0.0001) more resistant than younger mice carrying the reciprocal (sensitivity) alleles (MST = 111 h; n = 40), showing almost a 60% increase in survival time. Interestingly, mice with the sensitivity alleles for *Shali1–Shali2* showed no differences in MST based on age, with MSTs around 110 h for both age groups. Further parsing these groups by sex, we found significant differences between younger males (MST = 187 h; n = 20) with resistance *Shali1–Shali2* alleles compared to older males (MST = 127 h; n = 8) with resistance alleles (*p* = 0.0032), and also for younger males (MST = 125 h; n = 15) with sensitivity alleles for *Shali1–Shali2* compared to younger females (MST = 102 h; n = 25) with sensitivity alleles (*p* = 0.0067). Although not reaching significance (possibly due to low sample sizes), there was a trend of older females with either sensitivity (114 h *vs*. 106 h) or resistance (141 h *vs*. 127 h) alleles showing more HALI resistance than older males (Fig 4). This age- and sex-specific outcome for older females differed from that for total F_2_ mice, which found older males trended more resistant (Fig 2).

### Age and sex effects in congenic lines

To determine whether congenic lines carrying *Shali1* and *Shali2* also demonstrated the age- and sex-related effects, X1.B-1EA.a (sensitive) and X1.B-4BB (resistant) congenic lines of mice were exposed to hyperoxia and MSTs calculated (Table 2). Both sexes in all three age groups (≤ 6 weeks, 7–9 weeks, ≥ 10 weeks) spanning the 4-week trait transition period were tested and compared. The 7–9 week intermediate aged groups consistently gave intermediate MSTs, so were excluded from the table to better highlight the significant differences between the 6- and 10-week cohorts. Control X1 mice in each age group showed a slightly higher male resistance, similar to that seen in B controls. On average, the 10-week old X1 mice died about 3 days earlier than younger 6-week old X1 mice for both sexes. We reasoned that if a congenic line captured a gene(s) that imparts sensitivity (*i*.*e*., *Shali1*), then young *Shali1* congenic mice (which have the sensitivity alleles from the B strain) would die earlier than the normally resistant younger X1 mice. For both sexes, the MSTs of 6-week X1.B-1EA.a mice were significantly less than MSTs of young X1 mice (males: 142 h *vs*. 183 h; females: 94 h *vs*. 171 h), thus validating the capture of *Shali1* sensitivity in the X1.B-1EA.a congenic line. Of note, male X1.B-1EA.a congenics ≤ 6 weeks old were significantly less sensitive than age-matched females, indicating females would be much more informative for mapping the *Shali1* sensitivity locus. In fact, the MST of young X1.B-1EA.a females (94 h) was not significantly different than older X1 females (98 h), demonstrating that young X1.B-1EA.a congenic females have lost the usual resistance that exists in young X1 females (MST = 171 h).

**Table 2:** Age and sex differences in X1 controls *vs*. *Shali1* and *Shali2* congenic lines.

		Age at exposure (weeks ± 3 d.o.)					
Strain/Line	Sex	≤ 6 weeks (≤ 45 d.o.)			≥ 10 weeks (≥ 67 d.o.)		
		n	MST (h)	SD (h)	n	MST (h)	SD (h)
X1	M	14	183	36.0	73	114	11.3
(control)	F	15	171	51.2	23	98	12.5
X1.B–1EA.a	M	16	142*	43.4	6	79*	7.2
(*Shali1*)	F	9	94* ^,^ ^#^	21.9	4	77*	2.3
X1.B–4BB	M	12	215	18.1	16	186* ^,^ ^#^	33.0
(*Shali2*)	F	10	185	42.7	11	146*	38.2

* significantly different (*p* < 0.05) than age- and sex-matched X1 control

^#^ significantly different (*p* < 0.05) than age-matched opposite sex of same congenic line

Similarly, for *Shali2* to act as a resistance QTL, then *Shali2* congenics (which have the resistance alleles from the B strain) should have prolonged survival even at older ages, a time when control X1mice have become sensitive. As indicated in Table 2, males and females of older X1.B-4BB mice survived significantly longer than older X1 mice (186 h *vs*. 114 h, and 146 h *vs*. 98 h, respectively), and approached times of younger X1 mice. In this case, males averaged a survival time of about 40 h longer than females, indicating that males of this congenic line should be more informative for the overall resistant trait housed in *Shali2*.

A closer look at the MSTs of age-specific groups also provided evidence for an additive effect of *Shali1* and age on sensitivity, with older female X1.1EA.a mice even more sensitive than the younger X1.1EA.a congenics (77 h *vs*. 94 h) and older age-matched X1 mice (77 h *vs*. 98 h). Interestingly, older X1.1EA.a males totally lost the significant sex advantage seen in the younger male congenics (142 h *vs*. 79 h), suggesting this difference between male and female X1.1EA.a congenics could be exploited to identify a sex-related effect on sensitivity. For *Shali2*, the age effect (*i*.*e*., increased sensitivity of older X1.4BB mice) worked against the resistance trait in both sexes, but was especially apparent for X1.4BB females.

### Narrowing the *Shali1* interval

**Fig 5:**
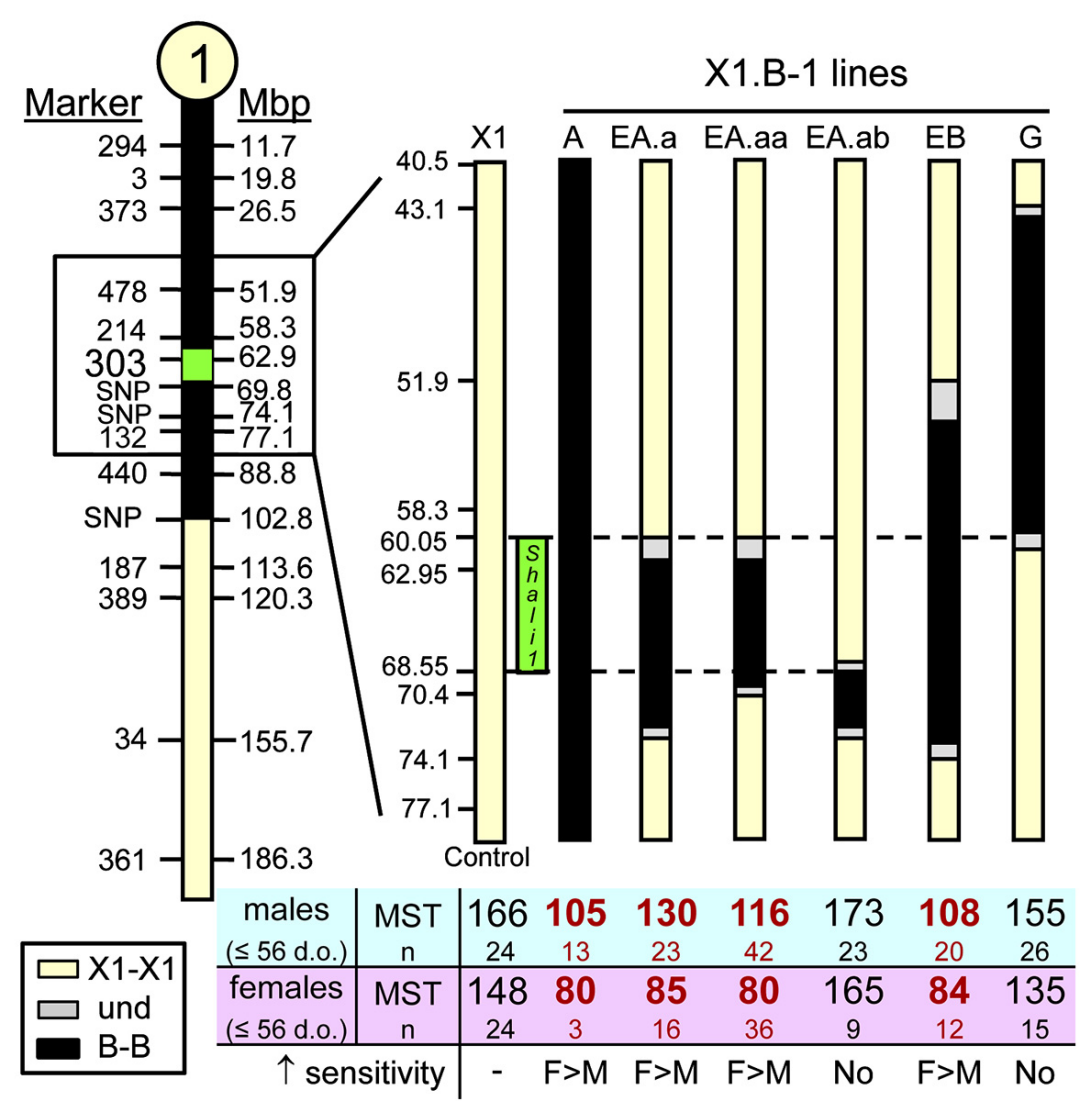
Sex-specific survival time comparisons of ≤ 56 d.o. *Shali1* congenic lines. Mice aged 56 d.o. and younger carrying different intervals of *Shali1* (boxed region on chromosome at the left) were exposed to >95% O_**2**_ and survival times determined and compared with age- and sex-matched X1 controls. A significant increase in sensitivity was determined in both males and females for four of six interval-specific congenics (bold **red** font), which narrowed the *Shali1* interval to ~8.5 Mb (as depicted by the dashed line and green box at 60.05–68.55 Mb). In all cases, females were also significantly more sensitive than males.

Our published data [7] demonstrated that both QTLs have strong effects on survival, endorsing the further refinement of these intervals. With the identified age effect, our focus for *Shali1* was now on younger highly sensitive mice, using a ≤ 56 d.o. age threshold to sort the data. Fig 5 displays the genetic regions captured in the *Shali1* congenic lines, along with separate MSTs for males and females. The original validated *Shali1* interval was 102.8 Mb (X1.B-1A; labeled: A), which encompassed slightly more than the proximal half of Chr1. The five subcongenics of the 1A interval have reduced this interval to ~8.5 Mb (60.05–68.55 Mb), as represented by the overlapping regions of the highly sensitive line 1EA.aa at 60–70.4 Mb and the congenic line 1EA.ab at ~68.5–74.1 Mb, which had a MST similar to control X1 mice. Of note, although both male and female 1EA.aa mice were significantly more sensitive than their age-matched X1 controls, females (MST = 80h) were strikingly more sensitive than males (MST = 116h), with the sensitivity trait showing almost full penetrance in X1.B-1EA.aa females of all ages tested. These data indicate that the causal gene(s) for HALI sensitivity within the *Shali1* interval can best be refined and ultimately identified using a comparison of young female X1.B-1EA.aa congenic mice and age-matched X1 females. In addition, this highly sensitive congenic (and the sub-congenic lines derived from it) can also be useful to distinguish the genetic and or hormonal factor(s) underlying the significant sex difference in younger *Shali1* mice (Table 1).

### Narrowing the *Shali2* interval

**Fig 6:**
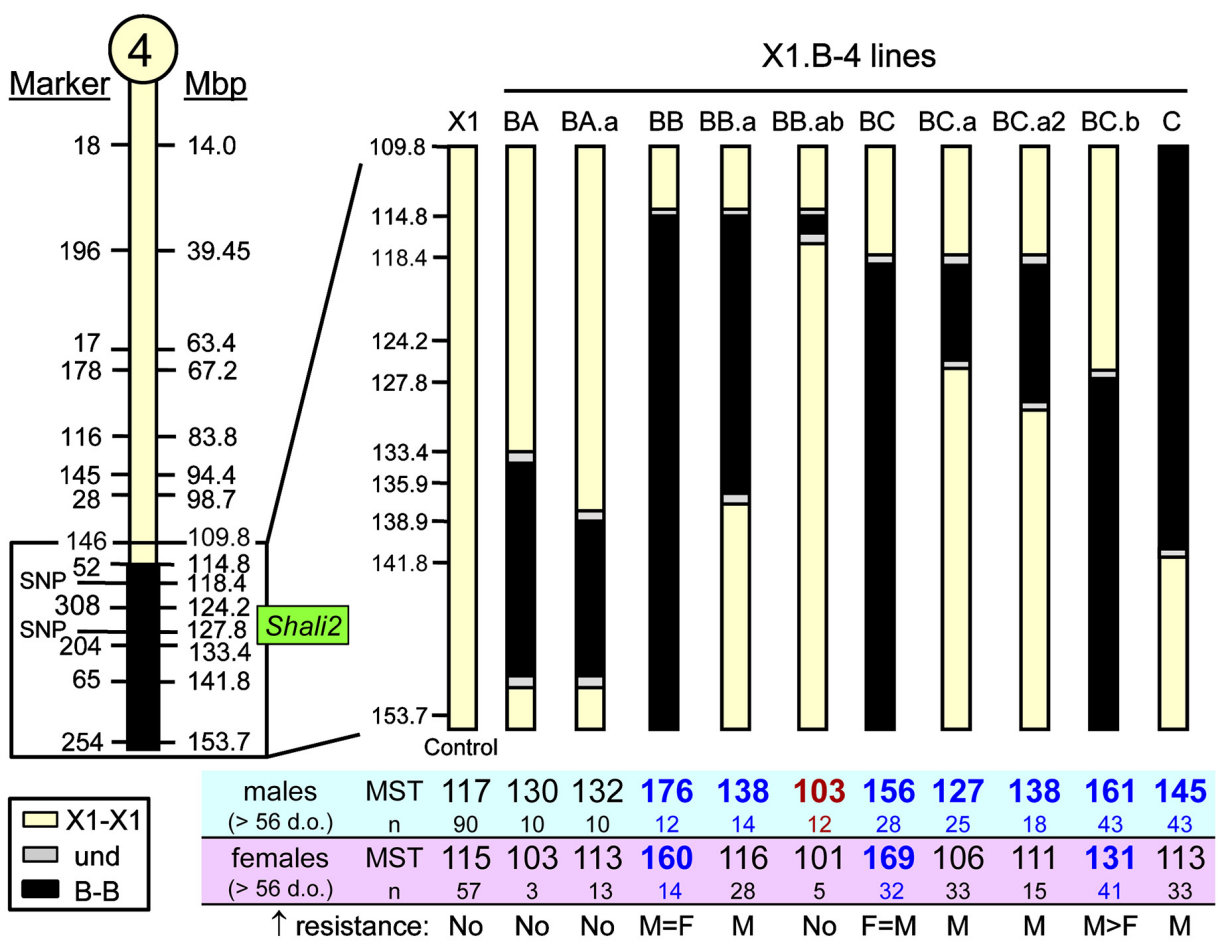
Sex-specific survival time comparisons of > 56 d.o. *Shali2* congenic lines. Mice > 56 d.o. carrying different subintervals of *Shali2* (boxed region on chromosome at the left) were exposed to > 95% O_**2**_ and survival times determined and compared with age- and sex-matched X1 controls. A significant increase in resistance was found in males only for four congenics and for both males and females for three congenics (bold **blue** font). One congenic (4BB.ab) was significantly more sensitive than controls for males (bold **red** font), with a trend for increased sensitivity in females. Congenics support multiple sub-QTLs in the *Shali2* interval.

Although *Shali1* appears to support a single genetic variant, *Shali2* is certainly more complex. Using the age and sex effect supported by the X1 controls and F_2_ population, we sought to narrow the *Shali2* interval with older mice (≥ 10 weeks) of relevant congenic lines (Fig 6). To date, the best overall resistance effect was captured in X1.B-4BB mice (labeled BB: ~114–155 Mb; 41 Mb) for both sexes. Male 4BB mice ≥ 10 weeks old were slightly more resistant (MST = 176 h) than age-matched females (MST = 160 h), with both sexes showing a similar penetrance of the resistance trait (*i*.*e*. 8/12 = 67% males *vs*. 8/14 = 57% females). Several subcongenic lines dividing the 4BB region have significant, but reduced, increases in MSTs, supporting multiple QTLs. Survival increases also varied for males and females among the different *Shali2* subcongenics, with males (*e*.*g*., X1.B-4BC.b) or females (*e*.*g*., X1.B-4BC) showing a longer MST. The smallest interval carrying the largest part of line 4BB increased resistance is contained within the congenic line 4BC.b (~127.8–155 Mb), which has ~75% of the increased survival of 4BB versus X1 males using the > 56 d.o. threshold (Fig 6). Importantly, at ≥ 10 weeks of age, males of the 4BC.b congenic line preserve most of this resistance (MST = 159 h, n = 24), whereas females of this congenic line have lost the resistance trait entirely at ≥ 10 weeks old, demonstrating a sensitivity (MST = 119 h, n = 26) similar to age-matched X1 mice (data not shown). These sex differences suggest that *Shali2* likely has male- and female-specific sub-QTLs that can be refined with ~10-week old mice of relevant congenic lines.

## Discussion

ALI is a serious clinical disease that can result from various direct or indirect injuries. Regardless of the cause, however, ALI is associated with high mortality. Differences in the occurrence, severity and outcome following an insult suggest that genetic factors are involved. Mortality rates for ALI also show a clear age-related pattern, ranging from > 60% in the elderly to 24% in patients 15–19 years of age [2] and 22% in pediatric patients [19]. This indicates that the young are more resistant and have better outcomes, whereas the elderly are highly susceptible, with advanced age being a strong predictor of ALI death [4–6,20]. ALI mortality is also age-dependent in rats and mice [21–23], as shown by the dramatic differential O_2_ tolerance of highly resistant newborn and young mice compared to the pronounced susceptibility of mature and older adults [24]. Yet, even though this age-dependent difference was reported decades ago, and has been confirmed by many investigators, exactly why maturity brings with it a loss of a protective response to oxidative lung injury is still unknown. It is within this realm of age-dependent O_2_ tolerance that our mouse models of differential HALI survival seem to fit.

Because ALI mortality is age-dependent, initial studies were designed to circumvent this complexity by using mice 6–12 weeks old; young adult mice in this narrow age window were anticipated to show similar HALI responses. However, we continued to see X1 inbred mice and X1-derived congenic lines fixed for *Shali1* or *Shali2* show large differences in survival times, even between litters from the same breeder pairs. This fluctuating penetrance of the resistance trait obligated us to explore alternative factors, such as age and sex that could explain the differential HALI survival in these lines. Since we had accrued a large dataset of exposed X1 mice over the years, the survival times could be sorted by age and sex into adequate-sized cohorts. When age at exposure was used to group X1 mice by sex into 1-week age bins, a remarkable shift in susceptibility was revealed, in which the MSTs of X1 mice changed from ‘highly resistant’ at 5–6 weeks old to ‘highly sensitive’ by 10 weeks of age (Fig 1). Even within the sensitive B strain, younger mice also had increased resistance over older mice, although this difference was modest in comparison. Interestingly, X1 mice at ≥ 10 weeks old had a MST similar to that for combined 6–12 week old B mice, demonstrating that the resistance trait in X1 mice has increased penetrance in younger mice. Once established in control strains, we next used three different strategies to evaluate the age-related survival effect in the large F_2_ dataset that originally identified the *Shali* QTLs [8]. Results of these analyses provided valuable age- and sex-specific relationships for *Shali1* and *Shali2* that were critical for advancing this project.

Using what was learned for age and sex to screen congenic lines of both QTLs, we have demonstrated large differences in survival times between age-matched males versus females in many different subcongenic lines. The reciprocal effects of the *Shali1* and *Shali2* congenics clearly showed that we have captured the different genetic factors representing the extremes of the trait shift (*e*.*g*., ‘resistant’ to ‘sensitive’) occurring in X1 mice between 6–10 weeks of age. In these congenics, penetrance was highest for sensitivity in 6-week old female *Shali1* mice (*i*.*e*., X1.B-1A), and highest for resistance in 10-week male *Shali2* mice (*i*.*e*., X1.B-4BB). Consistent with *Shali2* containing more than one QTL, data supports a possible female-specific resistance QTL at distal Chr4. Importantly, continued screening of congenics using age- and sex-relevant mice has reduced variability in survival times and, as seen for current congenic lines (Figs 5 and 6), should markedly enhance efforts to resolve these QTLs and identify the causal genetic factors.

The most refined *Shali1* congenic (X1.B-1EA.aa) has an interval of ~10.4 Mb. However, this sensitivity trait can be further reduced to a minimal region of ~8.5 Mb at 60–68.5 Mb on Chr1 by removing the overlap of congenic X1.B-1EA.ab (interval = ~68.5–74.1 Mb), which did not show sensitivity. *Shali1* appears to be a single gene or a small set of closely linked genes. The most refined single congenic that still carries a significant *Shali2* resistance effect (*i*.*e*., X1.B-4BC.b) has an interval of ~28 Mb (>126.7–154.9 Mb). This congenic has lost some of the overall resistance (especially in females) that was seen in the X1.B-4BB line, suggesting that additional sub-QTLs are present in the larger 4BB interval. Specific subcongenic lines of *Shali2* had a significant survival advantage for both females and males, yet others showed a survival advantage only in males. Interestingly, three congenic lines have maintained some increased resistance in females at 10 weeks old (*i*.*e*., 4BB, 4BC, and 4BC.b) and all three lines contain B alleles at the distal most portion of Chr4; the seven congenic lines without B alleles for this region have fully lost resistance in females. These 10 congenics support that a female-specific resistance locus maps to the distal end of Chr4, which was also identified by the separate QTL analyses of sex (Fig 3, top). In total, the data agree with multiple QTLs within the original *Shali2* interval, and suggest that they can be teased out by controlling for age and sex of corresponding congenic lines. These multiple sub-QTLs also closely correlate to the many peaks determined by the original F_2_ plot of *Shali2* [8] and the separate male and female QTL plots (Fig 3).

The exact status and developmental changes occurring in the lungs of B and X1 mice at 5–6 weeks of age are of critical importance to this age-related differential response, but such findings await additional detailed studies in control strains and the corresponding *Shali1* and *Shali2* congenic lines. Two biological processes—postnatal lung development and sexual maturation—are likely to be relevant in 5–6 week old mice. Although still unresolved, it is becoming increasingly clear that new alveoli continue to form into early adulthood in most mammals, including humans [25–27]. In particular, Mund *et al*. showed that alveolarization in mice proceeds until at least 36 days of age [26]. In humans, this period of developmental alveolarization usually occurs throughout the first 6–8 years of childhood, but maturation of the airways and lungs extends into adolescence [28,29]. Newer and likely more accurate estimates of postnatal human lung growth using stereological analysis [25] and Helium-3 magnetic resonance imaging [27] have shown that the number of alveoli increases through adolescence, a timeframe that correlates well with findings in mice [26], but not rats [25]. This timing also concurs with the age when X1 mice are still highly resistant to HALI but begin transitioning to a susceptible and then highly susceptible phenotype over the ensuing four weeks. In contrast, the B strain of mice is already sensitive by 6 weeks of age, suggesting that our X1-derived congenics, which carry B alleles for *Shali1*, should be a useful model to explore whether lung developmental and/or maturational differences exist between X1 and B, and whether these differences explain the survival time disparity in continuous hyperoxia exposures.

The age of mice with reliably high resistance to HALI (*i*.*e*., 5–6 weeks old) also overlaps the adolescence period. Besides residual lung maturation (as described above), the accompanying increases in sex hormones and sexual maturation occurring at this time add further complexities for identifying critical mechanisms at play. As sex hormones are also known to modulate a number of regulatory effects on lung development and maturation [30–32], it is likely that at least part of the sex differences identified in our lines of mice is due to hormonal effects on lung development. Indeed, it is common to see sex-specific differences for lung diseases and lung injuries that are also qualified by age-related differences. For example, adult male patients with ALI/ARDS have a significantly higher prevalence and higher mortality than females [2,33,34]. Likewise, male neonates have a higher risk of developing and dying from respiratory distress syndrome compared with female neonates [35]. We expect that our mouse models of differential HALI survival will be useful to examine these age- and sex-specific variables in detail for HALI and can be further tested for their applicability in other potentially relevant models of ALI.

In addition to ALI, sex differences in the incidence and pathogenicity of many lung diseases have been reported, including asthma, pulmonary fibrosis, and COPD [33,34,36,37]. Whereas females are more vulnerable to certain lung diseases, including lymphoangioleiomyomatosis [38], idiopathic pulmonary arterial hypertension [39], and connective tissue-associated interstitial lung diseases [40], males show a preponderance of idiopathic pulmonary fibrosis cases [37]. Examples of other sex-related complexities in lung disease include anatomic differences between the sexes [29], sex-specific polymorphisms associated with candidate genes [41], sex differences in lung metabolism [42], and age- and/or sex-specific gene expression differences [43,44]. Teasing out the underlying importance of sex and related sex hormones from genetic and environmental factors that also play significant roles in these lung diseases will remain challenging, but disease-specific mouse models like those reported herein for HALI will help us to target and control such parameters to better understand the separate and combined processes involved. Using such powerful mouse models, these sex differences can be further examined using pharmaceuticals, sex hormones, receptor inhibitors, removal of sex organs, gene knockdown, etc., to critically assess the possible cause and effects.

These HALI mouse models were developed as a strategy to discover important genes and pathways relevant to differential ALI/ARDS mortality. Towards that goal, the overall model has revealed significant age- and sex-related effects on HALI survival time in X1 and B strains, cohorts of the original F_2_ population generated from the B and X1 progenitor strains for QTL analysis, and the subsequent validated congenic and subcongenic lines for the major QTLs, *Shali1* and *Shali2*. We have provided evidence that the age-related effects on survival time in X1 mice correlated with the transition from high resistance at ~6 weeks of age to high sensitivity (similar to B-strain mice) by 10 weeks old. By transferring *Shali1* B alleles onto the X1 background (which already contains sensitivity X1 alleles at *Shali2*), we have generated a congenic line that is even more sensitive than B mice or older X1 mice (likely by additive effects). Similarly, by introducing *Shali2* B alleles onto the X1 background (which already contains *Shali1* resistance X1 alleles) we generated congenic mice that are able to maintain the resistance seen in younger mice for at least 3–4 additional weeks. Controlling for age has further strengthened these sensitivity and resistance traits by reducing variability of the response (due to increased penetrance), by boosting differences between age-specific X1 controls and congenics, and by improving the experimental design. Sorting by age also revealed large sex differences in several congenic lines of both QTLs, and highlighted that young females are best for refining the sensitivity trait of *Shali1*, but that *Shali2* contains sub-QTLs, which are male- or female-specific for increased resistance. These unique congenic lines will be valuable tools to (1) further resolve the *Shali1* and *Shali2* intervals and identify the corresponding age- and sex-specific causal factors involved in the differential susceptibility occurring around the transitional period of 6–10 weeks of age, (2) explore the separate physiologic and pathologic roles of *Shali1* and *Shali2* in ALI morbidity and mortality and (3) determine the important gene interactions affecting survival times in both directions. Further studies using these congenic lines with other mouse models of ALI, especially other oxidant-induced lung injuries, are practical and welcomed.
